# Germany's first Total Diet Study - Occurrence of non-dioxin-like polychlorinated biphenyls and polybrominated diphenyl ethers in foods

**DOI:** 10.1016/j.fochx.2024.101274

**Published:** 2024-03-11

**Authors:** Mandy Stadion, Katrin Blume, Christin Hackethal, Anja Lüth, David M. Schumacher, Oliver Lindtner, Irmela Sarvan

**Affiliations:** German Federal Institute for Risk Assessment (BfR), Max-Dohrn-Straße 8-10, 10589 Berlin, Germany

**Keywords:** BfR MEAL Study, Food, non-dioxin-like PCBs, Occurrence data, Organic and conventional type of production, PBDEs, Persistent organic pollutants, Total Diet Study, PCB 28 (PubChem CID: 23448), PCB 52 (PubChem CID: 37248), PCB 101 (PubChem CID: 37807), PCB 138 (PubChem CID: 37035), PCB 153 (PubChem CID: 37043), PCB 180 (PubChem CID: 37036), BDE-28 (PubChem CID: 12110098), BDE-47 (PubChem CID: 95170), BDE-49 (PubChem CID: 15509892), BDE-99 (PubChem CID: 36159), BDE-100 (PubChem CID: 154083), BDE-138 (PubChem CID: 15254861), BDE-153 (PubChem CID: 155166), BDE-154 (PubChem CID: 15509898), BDE-183 (PubChem CID: 15509899), BDE-209 (PubChem CID: 14410)

## Abstract

The occurrence of persistent organic pollutants like polychlorinated biphenyls (PCBs) and polybrominated diphenyl ethers (PBDEs) in food represents a public health concern. The BfR MEAL Study was initiated to generate a comprehensive data base of occurrence data for chemicals in the most consumed foods in Germany. Non-dioxin-like PCBs (NDL-PCBs) and PBDEs were analysed in 300 foods, purchased and prepared representatively for the eating behaviour of the population in Germany. Highest levels of NDL-PCBs and PBDEs were detected in spiny dogfish, cod liver, herring, and eel. High NDL-PCB and PBDE levels were observed in other oily fish, wild boar meat, sheep liver, and high-fat dairy products. The comparison of food from conventional and organic production revealed higher NDL-PCB values in the food group ‘meat and meat products’ if produced organically. Occurrence data of this study will improve future dietary exposure and risk assessments in Germany.

## Highlights

• Occurrence of NDL-PCBs and PBDEs in 300 foods prepared as consumed in Germany.
• Differentiation by region, season, and type of production in selected foods
• Highest NDL-PCB levels in fish products, fatty fish, mussels, and butter
• Highest PBDE levels in fish products, fatty fish, chocolate products, and wild boar

## 1 Introduction

Polychlorinated biphenyls (PCBs) and polybrominated diphenyl ethers (PBDEs) are aromatic compounds, classified as persistent organic pollutants (POPs). They are ubiquitous in the environment and can be detected in foods as well as in human tissues.

PCBs represent a group of 209 chlorinated congeners that differ in the number and position of chlorine atoms on the biphenyl backbone. Technical PCB mixtures were produced since the late 1920s and were used for various applications, mainly as non-flammable liquids with low conductivity in transformers and hydraulic systems (EFSA CONTAM, 2005). Like dioxins, PCBs are chemically stable and lipophilic; therefore, they are prone to accumulate in lipid-rich tissues and human breast milk. According to their toxicological properties, PCB congeners can be divided into two groups. Twelve congeners are known to induce toxicological effects similar to dioxins and are referred to as dioxin-like PCBs (DL-PCBs). All other PCB congeners display a different toxicological profile and form the group of non-dioxin-like PCBs (NDL-PCBs).

In animal studies conducted with individual NDL-PCB congeners, mainly effects on the thyroid and liver as well as on nervous, immune, endocrine and reproductive systems were discovered. Some of these findings were also observed in epidemiological studies including changes in thyroid hormones, neurodevelopmental and immunological effects, and some types of cancer (JECFA, 2016). Although many countries restricted the production and use of PCB mixtures in the 1980s and levels declined over the decades, NDL-PCBs are still released into the environment, largely via leaks or improper disposal practices (EFSA CONTAM, 2005). The sum of six NDL-PCB congeners is recommended to characterise the presence of NDL-PCBs as they represent about 50% of all PCB congeners in food and regulatory maximum levels, amounts that may not be exceeded, for different food categories were set in the European Union (Commission regulation (EU) 2023/915). However, a health-based guidance value has not yet been derived due to insufficient data.

Polybrominated diphenyl ethers (PBDEs) are a group of 209 organic compounds that were widely used as flame-retardants in the past. They are characterised by a diphenyl ether backbone and congeners differ in the number and position of the bromine substituents on both phenyl rings. For commercial use, technical PBDE mixtures (PentaBDE, OctaBDE, DecaBDE), each containing several congeners, were produced since the 1970s (La Guardia, Hale, & Harvey, 2006). To reduce combustibility, PBDEs have been added to a wide range of textile materials, foam upholstery, and plastics that were used in construction materials and numerous consumer products (e.g., furniture, mattresses, vehicles, electronic equipment). PBDEs are not chemically bonded to the materials and due to their low vapour pressure and lipophilic character, they can easily leach into the environment and bioaccumulate in the food web (Klinčić, Dvoršćak, Jagić, Mendaš, & Herceg Romanić, 2020). During the 1990s, the rising concentrations of PBDEs in the environment and in animal and human tissues gave rise to growing concern, especially due to a strong increase in human breast milk samples between 1972 and 1997 (EFSA CONTAM, 2011a).

After a detailed risk assessment for all commercial PBDE products under the EU Existing Substances Regulation (793/93/EEC), PentaBDE and OctaBDE were banned in the EU in 2004 (Directive 2003/11/EC) due to potential harm of the environment and for the protection of breastfed infants. Since then, the production and use of several further PBDE congeners have been regulated in the EU (EU REACH Regulation No. 1907/2006; EU POP Regulation No. 2019/1021). Since 2009, certain BDE mixtures are listed as POPs for elimination in Annex A of the Stockholm Convention.

Despite extensive restrictions and a noticeable decline in PBDE levels, they are still present in the environment and detectable in various foods (EFSA CONTAM, 2011a). Today, the dietary exposure to PBDEs in the European population is likely to raise a health concern (EFSA CONTAM, 2024). Multiple toxicological studies determined the liver, the thyroid hormone homeostasis as well as the immune, reproductive, and nervous system as main targets for negative health effects upon PBDE exposure. Maximum levels for PBDEs in food have not been established so far.

The amount of POPs in the local flora and fauna highly depends on the contamination levels in soil and sediments. The degree of pollution is thereby influenced by former use of the compounds in different commercial and industrial applications as well as by current emission sources, such as sealants, building materials, paints, and landfills (Weber et al., 2018). Specifically concerning PBDEs, e-waste recycling sites and plastic debris in marine ecosystems were identified as major sources for environmental contamination and future risks (EFSA CONTAM, 2024). It is described that PCB and PBDE levels in industrial areas exceed those in agricultural and rural areas (EFSA CONTAM, 2024; EFSA CONTAM, 2005). Consequently, the body burden of residents from areas with former production sites and disposal can be noticeably higher, as studies from Slovakia and North America confirmed (EFSA CONTAM, 2024; JECFA, 2016).

With regard to consumers health protection, a representative knowledge of the occurrence of undesirable substances in the diet of a population is crucial for reliable exposure and risk assessments. Total Diet Studies (TDSs) are the most cost-effective tool to address public health mission (EFSA/FAO/WHO, European Food Safety Authority, Food and Agriculture Organization of the United Nations, World Health Organization, 2011). The design of a TDS takes into account that levels of substances could decrease, increase or occur during industrial processing or food preparation at home. The BfR MEAL Study (meals for exposure assessment and analysis of foods) is the first German TDS (Sarvan, Bürgelt, Lindtner, & Greiner, 2017). It was implemented at the German Federal Institute for Risk Assessment (BfR, Berlin, Germany) and followed the guidelines of the European Food Safety Authority (EFSA), the Food and Agriculture Organization of the United Nations (FAO), and the World Health Organization (WHO) (EFSA/FAO/WHO, European Food Safety Authority, Food and Agriculture Organization of the United Nations, World Health Organization, 2011). In line with the three principles of a TDS, investigated foods were: i) representative for the entire diet consumed by the population in Germany, ii) prepared as consumed, and iii) subsequently pooled to one TDS food if related. In addition, for specific foods, we determined occurrence data between four regions, two seasons and type of production (conventionally and organically produced foods) to detect possible influences of these factors.

The aim of our research was to generate a comprehensive data base of NDL-PCB and PBDE levels in foods that were purchased and prepared representatively to consumers in Germany. Our study results will improve future dietary exposure assessments and thereby contribute to consumer health protection in Germany, to prevent possible health risks.

## 2 Material and methods

### 2.1 Food sampling

The BfR MEAL Study complies with the underlying principles of a TDS that were described in detail elsewhere (Sarvan et al., 2017). Food sample selection was based on a consumption survey among infants and young children (VELS, age 6 month up to 4 years) (Banasiak, Heseker, Sieke, Sommerfeld, & Vohmann, 2005) and 24-h dietary recall data from the second German National Nutrition Survey (NVS II, age groups 14–80 years) (Heuer, Krems, Moon, Brombach, & Hoffmann, 2015).

For the investigation of NDL-PCBs and PBDEs, foods where quantifiable results were expected were chosen via consulting an established expert group. Our results focus on foods of animal origin, cucurbits, and foods prepared with animal source ingredients, causing an adjusted, shorter food list compared to other substances analysed in the BfR MEAL Study. The 300 individual MEAL foods selected for the analysis of NDL-PCBs and PBDEs covered at least 90% of the average diet in Germany for different age groups. Additionally, the MEAL food list includes food items that are rarely consumed, but exhibited values around the EU maximum levels in food monitoring reports (e.g., eel, cod liver). MEAL foods were assigned to 17 main food groups in respect to the FoodEx2 classification. Similar food items such as pizza with salami and pizza with ham were pooled to one MEAL food (pizza with meat). For the intended coverage of four regions, two seasons and two types of production, some MEAL foods were purchased repeatedly. Therefore, one MEAL food could be represented by up to ten different MEAL food pools. MEAL foods with no regional differentiation were purchased in the area of Berlin and Brandenburg. MEAL foods sampled regionally (*n* = 51) were acquired in four regions (East, South, West, North) at three different shopping locations in each region (big city, small city, rural area). To integrate the influence of global food supply including variances in the exporting country, climate, soil, and animal husbandry conditions, some MEAL foods were sampled at two different times of the year (*n* = 38). After consultation with the International Advisory Board of the study, selected MEAL foods were differentiated into conventionally and organically produced food pools (*n* = 87).

All MEAL food pools with and without differentiation consisted of 15 and 20 single subsamples, respectively. Exceptions with less subsamples due to limited availability were the food pools boletus/porcino mushroom, edible offal (pork, beef), and maize germ oil. Pooled samples were combined on the basis of consumption data and market share data. Based on market share data from a representative panel with more than 30,000 households, subsamples were bought to represent the shopping behaviour of the population (e.g., shopping sites, brand, variety).

For the investigation of NDL-PCBs and PBDEs in 300 MEAL foods, 645 MEAL food pools were generated from 10,524 subsamples that were collected between December 2016 and May 2019.

### 2.2 Food preparation and homogenisation

In line with the principles of a TDS, food samples were processed and prepared as consumed before analysis. Subsamples were generated in the BfR study kitchen with the objective to imitate the typical preparation habits of the population in Germany. For this, representative telephone-based and online surveys were conducted to collect data on cooking methods, used kitchen utensils, preferred browning degrees, out-of-home consumption, and sources of recipes (Hackethal et al., 2023). In addition, the most visited cooking homepages and most sold recipe books were considered. For ingredients that made up less than 5% of a specific recipe (wet/weight), the most sold brand according to market share data was used (conventional production).

After preparation, all subsamples were pooled together to form one MEAL food pool and were homogenised using a knife mill (Grindomix GM300, Retsch GmbH, Haan, Germany). If required, ultrapure water (Milli-Q Integral 5, Merck, Darmstadt, Germany) or liquid nitrogen was added to achieve homogenisation.

As foods were prepared as consumers would do, all influences not reflecting typical consumer behaviour were excluded or minimized. Storage boxes that might release PBDEs were lined with strong aluminium foil to prohibit cross-contaminations. Samples were stored in amber glass vessels at −20 °C until analysis.

### 2.3 Analysis of NDL-PCBs and PBDEs

In this study, six NDL-PCB congeners (PCBs 28, 52, 101, 138, 153, and 180) were analysed. For the evaluation of PBDEs, the ten congeners BDE-28, −47, −49, −99, −100, −138, −153, −154, −183, and − 209 were selected as they are the most frequent congeners and included in the update of the risk assessment of PBDEs in food by the EFSA Panel on Contaminants in the Food Chain (CONTAM) (EFSA CONTAM, 2024).

NDL-PCB and PBDE levels in MEAL food pools were determined with an accredited gas chromatography/high resolution mass spectrometry (GC/HRMS) method (DIN EN ISO/IEC 17025) at the Chemical and Veterinary Analytical Institute Münsterland-Emscher-Lippe (CVUA-MEL, Münster, Germany) according to the criteria of Commission Regulation (EU) 2017/644 and the Guidance Document on the Determination of Organobromine Contaminants of the European Union Reference Laboratory for halogenated Persistent Organic Pollutants in Feed and Food (EURL POPs).

#### 2.3.1 Extraction and sample clean-up

Up to 100 g of a MEAL food pool homogenate was used for fat extraction. Used solvents (Picograde) were obtained from Promochem (Wesel, Germany). Cold extraction (dichloromethane/cyclohexane) was used for pasty matrices (e.g., eggs, meat, fish and liver), soxhlet extraction (toluene/acetone) for dry samples of plant origin and liquid/liquid extraction (ethanol/n-pentane) for milk samples. Extracts were evaporated to dryness with a rotary evaporator. Depending on the expected concentration, the complete extract or up to 4 g of fat per sample were dissolved in 10 ml of n-hexane. The clean-up of the samples was realised fully automatically on a DEXTech Plus sample preparation system (LCTech, Obertaufkirchen, Germany). Using ready-to-use LCTech columns (acid silica, alumina, and activated carbon), NDL-PCBs and PBDEs were flushed by a mixture of n-hexane/dichloromethane and collected in fraction 1.

Stable-isotope labelled internal standards (Wellington Laboratories, Guelph, Canada) were added before the extraction. ^13^C_12_-labelled recovery standards were added to the extracts prior to the measurement.

#### 2.3.2 GC/HRMS detection

Levels of NDL-PCBs and PBDEs were analysed by GC/HRMS using a Trace GC Ultra (Thermo Fisher Scientific, Waltham, USA)/DFS Magnetic Sector GC-HRMS System (Thermo Fisher Scientific) with a resolution of *R* = 10,000. NDL-PCBs were measured by injection of 2 μl splitless (280 °C) into a HT-8 capillary column (50 m, 0.22 mm, 0.25 μm film thickness). For the detection of PBDEs, 2 μl was injected splitless (275 °C) to a DB-5 capillary column (12 m, 0.20 mm, 0.11 μm film thickness). To confirm the reproducibility of the measurement (single determination), samples were repeated in regular intervals. A laboratory blank was included in each batch of seven samples and a quality control pool was included in every fifth batch.

For NDL-PCBs, the limit of quantification (LOQ) was determined for every analysed sample. LOQs ranged from 0.001 pg/g to 15 pg/g wet weight (ww) and from 0.013 pg/g to 22 pg/g fat. Depending on the congener and the sample matrix, the limit of detection (LOD) and LOQ of PBDEs ranged from 0.02 to 2 pg/g ww and from 0.06 to 5 pg/g ww, respectively.

#### 2.3.3 Data evaluation

Left-censored data were analysed using the upper bound (UB) scenario for NDL-PCBs and PBDEs. Results below LOD and LOQ were set to the value reported as LOD and LOQ, respectively. The modified lower bound (mLB) approach was applied for the sum of PBDEs in individual MEAL food pools as well, whereby results below LOD were replaced by zero and results below LOQ by the value reported as LOD.

Unless stated otherwise, results below are presented as mean UB levels of MEAL foods. For example, if a MEAL food was analysed in four different regions, the mean value of these four MEAL food pools was used for further evaluations and comparisons. Standard deviations determined on the main food group level reflect the variability between different MEAL foods in the particular group and do not consider the variation covered by single MEAL food pools or subsamples. In line with the design of a TDS, data from individual subsamples were not determined.

NDL-PCB and PBDE levels in MEAL foods were presented as the sum of six NDL-PCBs (∑6 NDL-PCBs) and the sum of nine or ten PBDEs (∑9 PBDEs, ∑10 PBDEs), respectively. Depending on the type of food, NDL-PCB values were either shown as ng/g ww or ng/g fat. In some tables and figures, all NDL-PCB levels were converted to ng/g ww for better comparison. All measured PBDE values were presented in pg/g ww.

If applicable, NDL-PCB occurrence data were compared with individual EU maximum levels, considering the measurement uncertainty.

## 3 Results and discussion

### 3.1 *NDL-PCB levels in MEAL foods*

In the BfR MEAL Study, NDL-PCBs were investigated in 645 MEAL food pools referring to 300 MEAL foods in 17 main food groups. Quantifiable levels were detected in nearly all food pools. Only in 14 samples (e.g., cornflakes, vegetarian sandwich spread, tofu), results for all six NDL-PCBs were < LOQ. Each of the congeners was quantifiable in 97% of the analysed food pools.

Highest mean UB levels for ∑6 NDL-PCBs were observed in food groups containing foods of animal origin. Highest values by far were detected in the food group ‘fish, seafood and invertebrates’ (12.4 ng/g ww), followed by ‘animal and vegetable fats and oils’, ‘meat and meat products’, and ‘milk and dairy products’ with 0.305, 0.167 and 0.157 ng/g ww, respectively (Table 1). Considering levels in foods of animal origin based on the fat content, the group ‘milk and dairy products’ showed highest NDL-PCB levels (1.44 ng/g fat) (Table S1). Levels in ‘animal fats’ (1.36 ng/g fat) and ‘meat and meat products’ (1.19 ng/g fat) were comparably high, the category ‘eggs and egg products’ (0.572 ng/g fat) exhibited slightly lower levels.

It is well known that food consumption is the main pathway of exposure to POPs like NDL-PCBs in humans (JECFA, 2016). As NDL-PCBs are lipophilic compounds that display a high absorption rate and biological half-life in the body, they tend to bioaccumulate in exposed animals and they also transfer into milk and eggs (JECFA, 2016; Ohlhoff et al., 2022). Our results confirmed high NDL-PCB levels in foods of animal origin with highest values detected in fish. Our data set is broadly consistent with TDS data from other European countries. In the second French and the UK TDS, highest NDL-PCB levels were also reported for the food group ‘fish’ with 5.26 and 2.30 ng/g wet weight, respectively (Fernandes, Rose, Smith, & Holland, 2012; Sirot et al., 2012), while we observed higher values. Differences in TDS data between countries can be due to differences in local contamination levels and food import strategies. Also varying consumption behaviours of the respective population would result in a different composition of the food list and likely explain the difference of the previous comparison. Our sampling covered a wide range of fish species, seafood and fish products with NDL-PCB levels ranging from 0.023 to 190 ng/g wet weight. The food list of the second French TDS did not include foods with outstanding high levels such as oily fish species, cod liver or high trophic species like spiny dogfish. However, comparing individual food pools such as Alaska pollock and salmon, levels reported in the second French TDS were slightly higher compared to those in the present study (ANSES, 2011).

**Table 1:** Mean levels of ∑6 NDL-PCBs in the main food groups in ng/g wet weight.

Main food group	MEAL foods (n)	Mean	SD	Median	Minimum	Maximum
Grains and grain-based products	38	0.047	0.046	0.030	0.002	0.206 (cream cake)
Vegetables and vegetable products	18	0.038	0.039	0.018	0.006	0.143 (kale)
Starchy roots or tubers and products thereof	7	0.029	0.044	0.009	0.003	0.124 (potatoes, pan-fried)
Legumes, nuts, oilseeds and spices	20	0.057	0.048	0.055	0.002	0.184 (spices)
Fruit and fruit products	8	0.012	0.008	0.010	0.003	0.026 (jam, fruits)
Meat and meat products^1^	35	0.167	0.206	0.106	0.004	0.926 (sheep liver)
Fish, seafood and invertebrates^2^	30	12.4	37.5	2.12	0.023	190 (spiny dogfish, smoked)
Milk and dairy products	23	0.157	0.180	0.055	0.012	0.682 (cream, plain)
Eggs and egg products	2	0.079	0.019	0.079	0.065	0.092 (eggs, pan-fried)
Sugar, confectionery and water-based sweet desserts	10	0.074	0.056	0.071	0.001	0.187 (milk chocolate)
Animal and vegetable fats and oils	8	0.305	0.454	0.089	0.016	1.18 (butter)
Coffee, cocoa, tea and infusions	7	0.017	0.028	0.003	0.001	0.076 (cocoa powder)
Alcoholic beverages	8	0.004	0.002	0.004	0.002	0.007 (spirit)
Food products for infants and toddlers	11	0.019	0.013	0.019	0.003	0.043 (ready-to-eat mixed meal)
Products for non-standard diets and food imitates	7	0.018	0.022	0.008	0.003	0.066 (vegetarian sausage)
Composite dishes	52	0.062	0.048	0.043	0.002	0.200 (sushi)
Seasoning, sauces and condiments	16	0.094	0.102	0.062	0.002	0.388 (cheese sauce)
Total/Mean	300	0.796	2.29	0.163		

Left censored-data were analysed using the upper bound scenario. Results below the limit of quantification (LOQ) were set to the value reported as the LOQ.

∑6 NDL-PCBs: PCB 28, 52, 101, 138, 153, 180.

1 Includes liver and edible offal.

2 Includes cod liver.

**Fig. 1:**
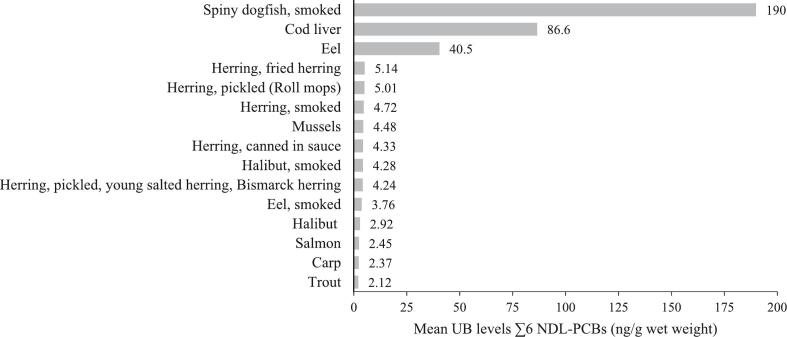
Mean levels of the sum of six NDL-PCBs in the 15 MEAL foods exhibiting the highest upper bound levels, expressed in ng/g wet weight.

Data of all individual MEAL food pools (∑6 NDL-PCBs and single congeners) can be found in Table S2 (ng/g ww) and Table S3 (ng/g fat). MEAL foods with the maximum value per food group are listed in Table 1. The 15 MEAL foods exhibiting the highest NDL-PCB levels are depicted in Fig. 1. All these foods belong to the main food group ‘fish, seafood and invertebrates’ with smoked spiny dogfish exhibiting the highest value by far (190 ng/g ww). Second and third highest levels were found in cod liver (86.6 ng/g ww) and eel (40.5 ng/g ww). High NDL-PCB levels were also measured in all five sampled herring products, mussels, smoked halibut, smoked eel, halibut, salmon, carp, and trout (Fig. 1). Fish with a high fat content mostly contained higher PCB levels than lean fish species like Alaska pollock and cod (Table S4, including all scientific names). In the foods of animal origin calculated relative to the fat content (54 MEAL foods), the highest ∑6 NDL-PCBs was detected in wild boar meat (11.8 ng/g fat) (Table S3) followed by yoghurt (2.75 ng/g fat) and condensed milk (2.45 ng/g fat). High levels were also found in meat from deer (2.41 ng/g fat) and sheep (2.36 ng/g fat) as well as in various dairy products. Comparing meat of different terrestrial animal species, game and sheep exhibited considerably higher values than bovine meat, pork meat or poultry (Table S3).

In 2010, EFSA published NDL-PCB monitoring data in foods collected from twenty countries between 1995 and 2008 (EFSA, 2010). Highest NDL-PCB levels were reported in the food groups ‘muscle meat eel’ (223 ng/g ww), ‘fish liver’ (148 ng/g ww), and ‘muscle meat fish and fish products excluding eel’ (23 ng/g ww). Remarkably, these mean UB levels were much higher than respective levels in the current study. This could be partly explained by the principal design of a TDS. In comparison to the analysis of samples on commodity level in national food monitoring programmes, TDS samples are prepared and analysed as consumed (EFSA/FAO/WHO, European Food Safety Authority, Food and Agriculture Organization of the United Nations, World Health Organization, 2011; Kolbaum et al., 2022). Hence, manufacturing processes, defrosting and preparation steps like trimming of fat and cooking and frying could lead to the reduction of NDL-PCB levels in processed foods (JECFA, 2016). Moreover, data submitted to EFSA were considerably older and might reflect the levels in a different time span. Lower levels in the BfR MEAL Study could be explained by a possible further decrease in background contamination levels over time, as shown for 1995 to 2010, as well as by a potential overestimation of left-censored data by the reporting countries due to higher analytical limits in the past (EFSA, 2012).

NDL-PCB levels in smoked spiny dogfish did not exceed the current EU maximum level for wild caught spiny dogfish. This level was increased from 75 to 200 ng/g ww in 2015 because many samples could not comply with the previous maximum levels under normal catch and growing conditions (Commission Regulation (EU) 2015/704). However, with 190 ng/g ww, single subsamples of the food pool might exceeded the maximum level as pooled samples per definition result in mean values.

The number of studies on the occurrence of NDL-PCBs in spiny dogfish is very limited. However, mean values observed in animals caught in southern New England and Florida coastal waters published by (Johnson-Restrepo, Kannan, Addink, & Adams, 2005) and (St-Gelais, Aeppli, Burnell, & Costa-Pierce, 2017) were more than 2- and 3-fold lower than in our study, respectively. This deviation could be explained by the region of catch, by the age and the length of the fish or most likely by the different fat contents of the investigated parts of the shark. In our study, we analysed smoked belly flaps with a comparably high fat content of 24% (Table S 4), whereas the other groups examined unprocessed muscle tissue (10% fat) or fillets (2% fat). Spiny dogfish is a species of higher trophic level with an average life span of 35 to 40 years. Comparatively high levels of POPs in this shark species can be explained by food-web transfer and biomagnification (St-Gelais et al., 2017). Besides to NDL-PCBs, spiny dogfish also exhibited high levels for methylmercury (Sarvan et al., 2021) and dioxins and dioxin-like PCBs (Stadion et al., 2022). Next to spiny dogfish, cod liver and eel represent foods that often exceeded their prescribed EU maximum levels (cod liver: 200 ng/g ww; eel: 300 ng/g ww) in national food monitoring (EFSA, 2012). However, in the current study, NDL-PCB levels were markedly lower.

With 0.926 ng/g ww, sheep liver exhibited the highest NDL-PCB level in the food group ‘meat and meat products’ (EU maximum level: 3.0 ng/g ww). Our result was similar to the mean value from data submitted by eight European countries to EFSA (EFSA CONTAM, 2011a). As the major detoxifying organ of the body, the liver is prone to deposit comparably high levels of POPs like dioxins and PCBs. In samples from sheep and cattle, NDL-PCB levels in liver were three times higher than in meat. Although not in Germany, a theoretically frequent consumption of sheep liver was described to represent a potential health concern, especially for children and woman of child-bearing age (EFSA CONTAM, 2011b).

**Fig. 2:**
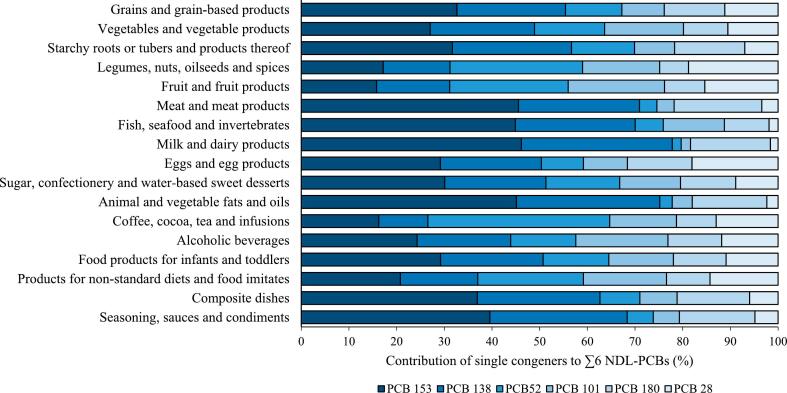
Mean upper bound relative contribution of NDL-PCBs across the 17 main food groups (300 MEAL foods).

The congener PCB 153 contributed mostly to the sum of NDL-PCBs in the 300 investigated MEAL foods (30%), followed by PCB 138 (21%), PCB 52 (14%), PCB 180 (12%), PCB 101 (12%), and PCB 28 (11%) (data not shown). A high contribution of PCB 153 was predominantly observed in food groups with foods from animal origin (Fig. 2). It accounted for 46% to ∑6 NDL-PCBs in ‘meat and meat products’ and ‘milk and dairy product’ and for 45% in ‘fish, seafood and invertebrates’ and ‘animal and vegetable fats and oils’. In contrast, in ‘fruit and fruit products’, ‘legumes, nuts, oilseeds and spices’, and ‘coffee, cocoa, tea and infusions’, PCB 153 accounted only for 16–17% to the total sum.

PCB-153 and PCB-138 were reported to be the dominating congeners in other studies as well (EFSA, 2010; Ohlhoff et al., 2022). Less chlorinated congeners like PCB 28 tend to adsorb less strongly to soil and exhibit shorter half-lives compared with higher chlorinated congeners like PCB 153 (EFSA, 2010). Furthermore, higher chlorinated congeners are less susceptible to metabolic conversion resulting in an elevated potential for biomagnification along the food chain. Consequently, congener patterns can change from feed to food and congener-specific transfers can result in the enrichment of individual congeners in meat, milk, and eggs (Ohlhoff et al., 2022).

### 3.2 PBDE levels in MEAL foods

Levels of ten selected PBDE congeners were investigated in 645 MEAL food pools referring to 300 MEAL foods in 17 main food groups. PBDE levels were quantifiable in about 93% of the food pools. In 46 samples (e.g., banana, coffee, tofu), all observed PBDE levels were < LOD/<LOQ. BDE-28, −47, −49, −99, −100, −138, −153, −154, −183, and − 209 were quantified (values >LOQ) in 6, 82, 5, 83, 31, 2, 37, 19, 34, and 85% of the analysed food pools, respectively. The three congeners BDE-47, −99, −209 were detected in foods from all food groups. For BDE-28 and -49, quantifiable results were almost exclusively found in ‘fish, seafood and invertebrates’. BDE-209 was the most abundant congener in every food group (31–96%), except for ‘fish, seafood and invertebrates’ (2%) (Fig. S1 A). Considering the 300 MEAL foods, it contributed to 59% to the total sum (∑10 PBDEs), followed by BDE-47 (13%), and BDE-99 (11%) (data not shown). Due to this comparatively high contribution of BDE-209 and its deviating analytical performance criteria (EURL POPs), the dataset is additionally presented as ∑9 PBDEs (without BDE-209). According to the EURL Guidance Document, the targeted LOQ for each congener is expected to be below 0.001 μg/kg, except for BDE-209 (0.01 μg/kg). MEAL foods with the highest level of BDE-209 are depicted in Fig. S1 B. Results of individual congeners and ∑10 PBDEs of each MEAL food pool can be found in Table S5.

Highest mean UB values of ∑9 PBDEs were measured in food groups containing foods of animal origin. The main food group ‘fish, seafood and invertebrates’ exhibited the highest mean level by far (782 pg/g ww) followed by ‘meat and meat products’ (37.5 pg/g ww) (Table 2). ∑9 PBDE levels found in the categories ‘animal and vegetable fats and oils’ (21.0 pg/g wet weight), ‘eggs and egg products’ (18.3 pg/g wet weight), and ‘milk and dairy products’ (13.2 pg/g wet weight) were similar but markedly lower than those in ‘fish, seafood and invertebrates’.

**Table 2:** Mean levels ∑9 PBDEs in the main food groups in pg/g wet weight.

Main food group	MEAL foods (n)	Mean	SD	Median	Minimum	Maximum
Grains and grain-based products	38	8.34	10.2	6.90	2.12	65.2 (biscuits)
Vegetables and vegetable products	18	3.94	2.07	3.07	1.87	9.26 (kale)
Starchy roots or tubers and products thereof	7	3.92	2.59	3.67	1.44	9.15 (potato crisps)
Legumes, nuts, oilseeds and spices	20	6.61	4.82	4.98	2.31	17.9 (lentils)
Fruit and fruit products	8	2.97	1.95	2.34	1.95	7.76 (jam, fruits)
Meat and meat products^1^	35	37.5	46.7	15.0	3.47	184 (wild boar meat)
Fish, seafood and invertebrates^2^	30	782	2317	155	4.22	11,122 (spiny dogfish, smoked)
Milk and dairy products	23	13.2	11.6	10.3	2.12	41.2 (cream, plain)
Eggs and egg products	2	18.3	7.20	18.3	13.18	23.4 (hen egg)
Sugar, confectionery and water-based sweet desserts	10	6.31	4.23	5.78	1.44	13.2 (milk chocolate)
Animal and vegetable fats and oils	8	21.0	20.6	10.7	3.88	61.4 (butter)
Coffee, cocoa, tea and infusions	7	4.18	2.99	3.82	1.44	8.84 (rooibos tea, prepared)
Alcoholic beverages	8	1.50	1.62	0.878	0.580	5.40 (mixed alcoholic drink)
Food products for infants and toddlers	11	3.43	2.30	3.09	0.458	7.38 (infant formulae)
Products for non-standard diets and food imitates	7	3.11	1.80	2.12	1.44	5.95 (vegetarian sausage)
Composite dishes	52	10.3	15.1	6.30	1.78	107 (meatball)
Seasoning, sauces and condiments	16	6.84	5.26	6.70	0.660	14.9 (white/bright sauce)
Total/Mean	300	54.9	145	15.2		

Left censored-data were analysed using the upper bound scenario. Results below the limit of detection (LOD) were set to the value reported as LOD. Results below the limit of quantification (LOQ) were set to the value reported as LOQ.

∑9 PBDEs: BDE-28, 47, 49, 99, 100, 138, 153, 154, 183.

1 Includes liver and edible offal.

2 Includes cod liver.

Our data confirm highest PBDE levels in foods of animal origin, especially in those exhibiting a high fat content. The dietary uptake of PBDEs via animal source foods represents the major route of human exposure to these brominated flame retardants. Moreover, the inhalation and ingestion of PBDEs through indoor dust was described to essentially contribute to PBDE body burdens (primary source of BDE-209), especially in young children (Bramwell et al., 2017; Klinčić et al., 2020).

Our analysis revealed highest PBDE levels in fish liver and fish species rich in fat followed by meat and butter. It has to be noted that the direct comparison of occurrence data from different studies is often difficult due to the lack of consistency in the reporting of PBDE levels (e.g., number and kind of investigated congeners and foods, expression of the results). Recently, the EFSA published PBDE monitoring data from thirteen European countries reported from 2010 to 2019 (EFSA CONTAM, 2024) with noticeably higher levels compared to the present study. Reductions during processing and cooking as well as the time point of analysis could explain these divergences, but also geographical differences in environmental PBDE pollution could play a substantial role (EFSA CONTAM, 2024). In the second French TDS, Rivière and colleagues investigated eight PBDE congeners in 77 foods (Rivière et al., 2014). Their results were broadly consistent with our data, except for 6- and 8-fold higher values in the food groups ‘margarines’ and ‘vegetables’ compared to our study, respectively. PBDE levels determined in the UK TDS were, in some food groups, markedly higher than in our study, predominantly BDE-209 levels (Fernandes et al., 2012). This is corroborated with literature describing higher PBDE human body burdens and BDE-209 concentrations in indoor dust in the UK compared with other European countries. These findings seem to directly correlate with an intense historical PBDE usage in the UK that resulted from very strict fire protection regulations (Bramwell et al., 2017; Klinčić et al., 2020). Comparing our data with more recent occurrence data from Latvia showed similar PBDE levels in most of the food groups (Zacs et al., 2021).

**Fig. 3:**
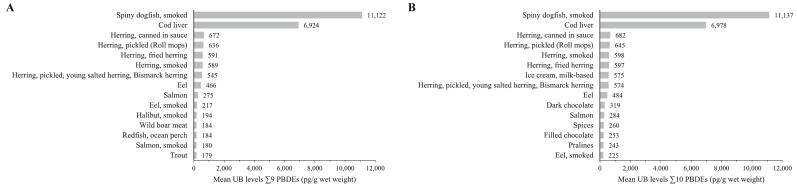
Mean levels of the sum of (A) nine PBDE congeners (without BDE-209) and (B) ten PBDE congeners (with BDE-209) in the 15 MEAL foods exhibiting the highest UB (upper bound) levels, expressed in pg/g wet weight.

Among all analysed MEAL foods, the highest ∑9 PBDE level was identified in smoked spiny dogfish with 11,122 pg/g ww (Fig. 3A). Cod liver showed the second highest level (6924 pg/g ww), followed by five herring products, eel, salmon, and other fish species. ∑9 PBDE and ∑10 PBDE levels for all MEAL foods from the category ‘fish, seafood and invertebrates’ are listed in Table S4. Next to fish and fish products, a comparably high value was determined in wild boar meat (184 pg/g ww).

Mean PBDE levels in spiny dogfish were about 3-fold higher compared with the result published by scientists from the USA (3780 pg/g ww) (Johnson-Restrepo et al., 2005). For cod liver, the mean PBDE value was twice as high as those from the Latvian monitoring (3484 pg/g ww) (Zacs et al., 2021), but 2.5-fold lower than the mean value reported by EFSA (17,392 pg/g ww) (EFSA CONTAM, 2024). The occurrence of numerous POPs in marine fish is strongly influenced by the catching area. Levels measured in food webs from the Baltic Sea were often higher compared with those in more northern and less populated and industrialized marine areas (Karl et al., 2016). The BfR MEAL study also reported high levels of dioxins (Stadion et al., 2022), cadmium (Fechner et al., 2022), and arsenic (Hackethal, Kopp, Sarvan, Schwerdtle, & Lindtner, 2021) in cod liver. However, it is also known for its high values of beneficial omega-3 fatty acids, vitamin A, vitamin D, and iodine (Linday, 2010; Schendel et al., 2022).

In the food group ‘meat and meat products’, wild boar meat displayed the highest PBDE level (Fig. 3A). The value was more than ten times higher compared with values detected in meat from deer, sheep, and cattle (Table S5). Being mainly wild-living omnivores, wild boars are more prone to take up and bioaccumulate POPs compared with herbivores and farmed animals. Due to their rooting behaviour, wild boars also tend to ingest comparatively high amounts of soil that is a major source of PBDEs (Weber et al., 2018). Publicly accessible data on the occurrence of PBDEs in wild boar meat are scarce. Levels observed is the present study were more than 10-fold higher than the mean value measured in individuals from Latvian wildlife (Zacs, Rjabova, Ikkere, Bavrins, & Bartkevics, 2018). The rather young average age of the specimens (1.4 years) as well as the low density of urbanisation and industrialization of the local hunting regions in Latvia might be a reason. Remarkably, in some of the subsamples in the BfR MEAL Study, the meat originally came from Australia and was sold in German supermarkets. As background PBDE levels in Australia are described to be markedly higher than those in Europe, this might explain the higher PBDE amount observed in our food pool as well (McGrath, Ball, & Clarke, 2017).

The consumption of foods higher up the food chain such as foods of animal origin represents the main source for PBDE exposure (Bramwell et al., 2017). Consequently, it can be assumed that the dietary exposure to PBDEs for people following a vegetarian or vegan diet is lower than that for people consuming an omnivorous diet (EFSA CONTAM, 2024). PBDE levels in foods of plant origin submitted to the EFSA were often below the LOQ. In contrast to these monitoring data, the BfR MEAL study obtained many detectable PBDE level in foods of plant origin. This might be explained by food preparation steps that included the addition of animal or vegetable fats and oils.

In Fig. 3B, results are presented as ∑10 PBDEs (including BDE-209). In contrast to the 15 foods with the highest concentrations of ∑9 PBDEs (Fig. 3A), MEAL foods other than fish and meat namely ice cream, dark chocolate, spices, filled chocolate, and pralines are listed. In these foods, BDE-209 represented the most prevalent congener providing 93–97% to the total sum (Fig. S1 B). Strikingly, many cocoa-containing MEAL foods showed very high PBDE values with BDE-209 as the dominating congener (Table S5). High occurrence of PBDEs in chocolate was also described in a recent publication (Zacs et al., 2021). Considering the low levels of BDE-209 in cow milk, it seems very likely that the values are driven by cocoa powder as an ingredient.

**Fig. 4:**
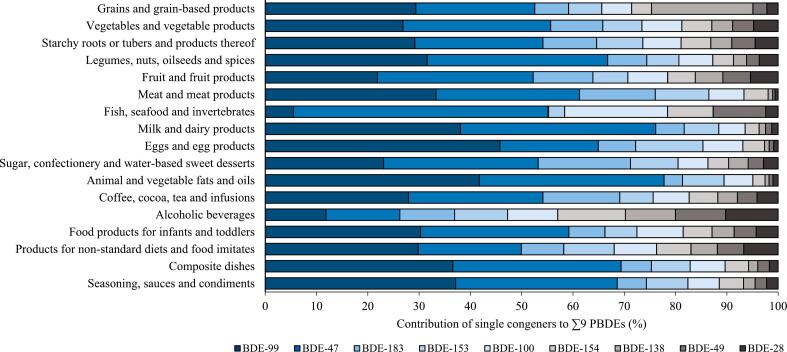
Mean upper bound relative contribution of PBDE congeners across the 17 main food groups (300 MEAL foods).

The predominant contributors to ∑9 PBDEs in MEAL foods were BDE-47 and -99 with a contribution of 29% and 28% to the total sum, respectively (data not shown). Looking at the main food groups individually, congener patterns differed (Fig. 4). With the exception of ‘alcoholic beverages’, BDE-47 and -99 together provided more than 50% to ∑9 PBDEs in every main food group. The PBDE congener profile in ‘fish, seafood and invertebrates’ differed considerably from those of the other food groups. This category showed the highest percentage of BDE-47, −49, and − 100 and the lowest of BDE-99, −138, −153, and − 183.

Congener profiles across the main food groups were comparable with those reported in literature. Mostly, they were dominated by the deca-brominated congener BDE-209, followed by the lower brominated congeners BDE-47 and BDE-99. The low presence of BDE-209 in fish species was also observed by others and can be explained by its very low potential for bioaccumulation within the marine food web (EFSA CONTAM, 2024). Reported ratios of BDE-47/99 found in soil, plants, and foods were approx. 1, reflecting their ratio of almost 1:1 in former penta-BDE commercial formulations (La Guardia et al., 2006). The levels of BDE-47 und BDE-99 were similar in most of the main food groups in the present study as well. One exception was the category ‘fish, seafood and invertebrates’ with strikingly lower contribution of BDE-99. Consistently higher BDE-47 levels compared with BDE-99 levels in fish species were also reported by other groups (EFSA CONTAM, 2024). It was suggested that this observation is partly caused by a less rapid uptake and a higher metabolism and excretion of BDE-99 in aquatic organisms. Moreover, biotransformation of BDE-99 to BDE-47 in fish was described in some experiments (Streets et al., 2006).

### 3.3 Impact of the region and season on NDL-PCB and PBDE levels

Fifty-one MEAL foods assigned to six main food groups were sampled in four different regions of Germany. In Table S6 and Table S7, mean NDL-PCB and PBDE levels differentiated by region are summarised, respectively. In total, mean values of examined food groups were not significantly different when comparing the four regions. Differences between specific foods could be found, but no pattern was observed.

Thirty-eight MEAL foods out of seven main food groups were purchased at two different seasons. Overall, mean NDL-PCB levels were comparable between season 1 and season 2 in the investigated foods groups (data not shown). However, some deviations in individual MEAL foods were observed (Table S2 and Table S3).

Regarding data on PBDEs, the comparison at the level of food groups showed similar results for both seasons (data not shown). No remarkable differences were found at the level of single MEAL foods (Table S5).

### 3.4 Impact of the type of production on NDL-PCB and PBDE levels

Eighty-seven MEAL foods out of 15 main food groups were sampled according to type of production. For NDL-PCBs, there was no clear effect across all food groups. Looking at the groups independently, the biggest difference between conventionally and organically produced foods was observed in ‘meat and meat products’ (*n* = 12, including animal livers) with 2.6-fold higher NDL-PCBs in organic samples (Fig. S2 A). In line with this, some meat-containing MEAL foods from the category ‘composite dishes’ (e.g., beef roulade, goulash, meat ball) showed higher NDL-PCBs if produced organically (Table S2). In ‘vegetables and vegetable products’, six out of seven MEAL foods exhibited lower values in the corresponding organically produced food pool (Fig. S2 A). For example, organic asparagus, carrot, and red cabbage displayed 5, 2.7, and 2.4 times lower NDL-PCBs than the corresponding conventional variants, respectively (Table S2).

The comparison of NDL-PCB levels in animal source foods expressed relative to the fat content (*n* = 19) revealed markedly lower levels in conventionally produced food pools (Fig. S2 B). The fat contents of the compared food pools were similar. The difference was mainly attributed to a 2.3-fold lower mean value in the food group ‘meat and meat products’ (*n* = 9, without animal livers). Differences in the categories ‘milk and dairy products’, ‘eggs and egg products’, and ‘butter’ were rather low and not significant (Fig. S2 B). MEAL foods with the greatest differences between the two types of production were salami-type sausage (pork, beef), liver sausage (pig, beef), and duck meat with 6.1, 4.1, and 3.4 times lower values in conventionally produced food pools, respectively (Fig. S2 C). NDL-PCB levels in hen eggs from organic production were 1.5-fold higher than from conventional production.

Regarding PBDE levels in main food groups, ∑10 PBDEs and ∑9 PBDEs were mainly comparable between the two types of production (Fig. S3 A and S3 B). The small non-significant difference in ‘meat and meat products’ was primarily caused by strongly deviating PBDE levels in the MEAL food pools fine cooked sausage (e.g. Lyoner, Mortadella) (conventional) and pork meat (conventional, North) (Table S5). Similarly, differences in the food groups ‘sugar, confectionery and water-based sweet desserts’ and ‘composite dishes’ were due to markedly higher PBDE levels in only one MEAL food pool each (conventionally produced milk chocolate and organically produced meat ball) (Table S5).

The type of production can have an impact on the levels of POPs in foods. However, there is no clear scientific evidence that organic foods are more nutritious or can lead to better health effects (González, Marquès, Nadal, & Domingo, 2019). As environmental pollutants may accumulate in soil and most foods, also organically produced food is not exempted from this risk. Data from the current study demonstrated higher NDL-PCB levels in a variety of organically produced foods of animal origin. Recently, we also reported on higher dioxin and DL-PCB levels in organically produced foods of animal origin (Stadion et al., 2022). Consistent with our observations, levels of dioxins, DL-PCBs, and NDL-PCBs were significantly higher in the organic variants of meat from France (Dervilly-Pinel et al., 2017).

Many studies that evaluated the impact of the type of production on the levels of POPs investigated a small selection of foods, often only hen eggs. Available research data on PBDE levels are scarce. Organic farming guarantees organic feed, more space per animal as well as outdoor access. The main exposure pathway for livestock animals with outdoor access is the uptake of soil, followed by feed, bedding, and housing materials (Pajurek, Pietron, Maszewski, Mikolajczyk, & Piskorska-Pliszczynska, 2019). Depending on the species, conventional animal husbandry can include the access to free-range areas as well. Therefore, the amount of time spent outdoors is considered to be the most relevant factor for differences in levels of POPs rather than feeding organic products (Roszko, Szymczyk, & Jędrzejczak, 2014; Weber et al., 2018). Comparing a number of studies, differences in eggs from different housing conditions were more pronounced for PCBs than for PBDEs (Pajurek et al., 2019; Roszko et al., 2014). Highest levels were found in the organic group followed by free range, barn, and cage (Pajurek et al., 2019). In the current work, NDL-PCB and PBDE levels were also higher in organically produced hen eggs, but the differences were rather small. One explanation could be that the conventional pool included free-range eggs and excluded eggs from cage farming.

### 3.5 Limitations and uncertainties of the study

The BfR MEAL Study is one of the most comprehensive TDS worldwide with a rather low aggregation level. Nonetheless, information on levels in single subsamples (e.g., variety, brand, preparation method) are not available due to pooling.

The MEAL food list covers more than 90% of foods consumed in Germany. The MEAL food list is based on consumption data from the VELS (2002) and the NVS II study (2006) covering data from children aged between 6 months up to 4 years and adults from 14 to 80 years, respectively. Hence, consumption habits of newborns and infants till the age of 6 month and of children in the age group 5 to 13 years were not considered. The assessment of the eating behaviour was based on two 3-day dietary records (VELS) or two 24 h-recalls (NVS II) on non-consecutive days. Rarely consumed foods might be underrepresented as well as foods frequently consumed by specific population groups (e.g., hunters, people following a vegetarian or vegan diet). As eating behaviours changed during the last years, as demonstrated for the German situation, current food trends might not be considered (e.g., oat milk, plant-based meat substitutes) (Kolbaum, Ptok, Jung, Libuda, & Lindtner, 2023).

Usage of the mLB and UB approach for left-censored data involves the possibility for under or over interpretation of actual levels, respectively. Differences between individual MEAL foods or MEAL food pools were stated descriptively. The sampling of the BfR MEAL Study was not designed to support specific hypothesis testing. Group comparisons were therefore only carried out for selected operations and can only serve as an indication of possible differences, while a systematic statistical analysis of explanatory factors for occurrence levels is outside the scope of the study.

## 4 Conclusion

In the scope of the BfR MEAL Study, we investigated 300 foods for the occurrence of six NDL-PCB and ten PBDE congeners, thus providing a comprehensive data basis for dietary exposure assessments and comparisons with other countries. As expected, highest mean levels were found in foods of animal origin, predominantly in the main food group ‘fish, seafood and invertebrates’. Spiny dogfish, cod liver, eel, and herring represented by far the MEAL foods with the highest NDL-PCBs and PBDE levels. But mean values did not exceed existing EU maximum levels. However, single subsamples may have exceeded the maximum levels. Comparatively high values were also detected in other fish species, cocoa-containing products (PBDEs), butter, sheep liver (NDL-PCBs), wild boar meat, and high-fat dairy products. For NDL-PCBs and PBDEs, PCB 153 and 138 and BDE-209 and -47 were the dominating congeners, respectively. The comparison of occurrence data between four regions and two seasons in selected MEAL foods revealed no major differences. Differentiating the type of production, values in foods of animal origin that were produced organically tend to be higher compared with the respective conventional products. This effect was mainly attributed to differences in the food group ‘meat and meat products’ and observed for NDL-PCBs only.

The current work generated a comprehensive data set on NDL-PCB and PBDE levels in the most consumed foods representative for the population in Germany. Hence, our data are crucial to reduce uncertainties in exposure assessments for the population in Germany which are caused by missing information on levels in several foods. TDS provide an important basis for the refinement of long-term dietary exposure assessments and thus contribute substantially to consumer health protection.

The following are the supplementary data related to this article.

Fig. S1: (A) Mean upper bound relative contribution of ten selected PBDEs across the 17 main food groups (300 MEAL foods). (B) BDE-209 in the 15 MEAL foods exhibiting the highest upper bound (UB) levels. Fig. S1

Fig. S2: Mean upper bound levels of the sum of six NDL-PCBs in MEAL food groups according to conventional and organic type of production based on the wet weight in (A) and on the fat content in (B). (C) Comparison of foods of animal origin by the type of production. 1 incudes liver samples. Data are means (n reflects the number of MEAL foods). Fig. S2

Fig. S3: (A) Mean upper bound (UB) levels of the sum of nine PBDEs (without BDE-209) in MEAL food groups according to conventional and organic type of production and (B) mean UB levels of the sum of ten PBDEs (including BDE-209). Data are means (n reflects the number of MEAL foods). Fig. S3

Table S1: Mean levels of ∑6 NDL-PCBs in main food groups with foods of animal origin expressed in ng/g fat. Table S1

Table S2: Differentiation of MEAL food pools with respective levels of ∑6 NDL-PCBs as well as levels of the single PCB congeners in ng/g wet weight. Table S2

Table S3: Differentiation of MEAL food pools with respective levels of ∑6 NDL-PCBs as well as levels of the single PCB congeners in ng/g fat. Table S3

Table S4: Mean upper bound levels of ∑6 NDL-PCBs, ∑9 PBDEs, ∑10 PBDEs as well as the fat content of MEAL foods from the main food group 'fish, seafood and invertebrates'. Table S4

Table S5: Differentiation of MEAL food pools with respective upper bound levels of single congeners as well as modified lower bound and upper bound levels of ∑10 PBDEs and ∑9 PBDEs (without BDE-209) in pg/g wet weight. Table S5

Table S6: Mean levels of ∑6 NDL-PCBs in the main food groups with MEAL foods that were analysed for differences in the region (ng/g wet weight). Table S6

Table S7: Mean levels of ∑9 PBDEs and ∑10 PBDEs in main food groups with MEAL foods that were analysed for differences in the region (pg/g wet weight). Table S7

## CRediT authorship contribution statement

**Mandy Stadion:** Writing – original draft, Visualization, Validation, Investigation, Formal analysis. **Katrin Blume:** Writing – review & editing, Validation. **Christin Hackethal:** Writing – review & editing, Methodology. **Anja Lüth:** Writing – review & editing, Writing – original draft, Validation. **David M. Schumacher:** Writing – review & editing, Validation. **Oliver Lindtner:** Writing – review & editing, Project administration, Funding acquisition, Conceptualization. **Irmela Sarvan:** Writing – review & editing, Supervision, Project administration, Methodology, Conceptualization.

## Declaration of competing interest

The authors declare that they have no known competing financial interests or personal relationships that could have appeared to influence the work reported in this paper.

## Data Availability

Data will be made available on request.
