# Supplementary material for: Germany's first Total Diet Study - Occurrence of non-dioxin-like polychlorinated biphenyls and polybrominated diphenyl ethers in foods
Source: Food Chem X. 2024 Mar 11;22:101274. doi: 10.1016/j.fochx.2024.101274 (PMC10957405; doi:10.1016/j.fochx.2024.101274)
Supplement: Fig. S1: (A) Mean upper bound relative contribution of ten selected PBDEs across the 17 main food groups (300 MEAL foods). (B) BDE-209 in the 15 MEAL foods exhibiting the highest upper bound (UB) levels. [file mmc1.pptx]

## Slide 1
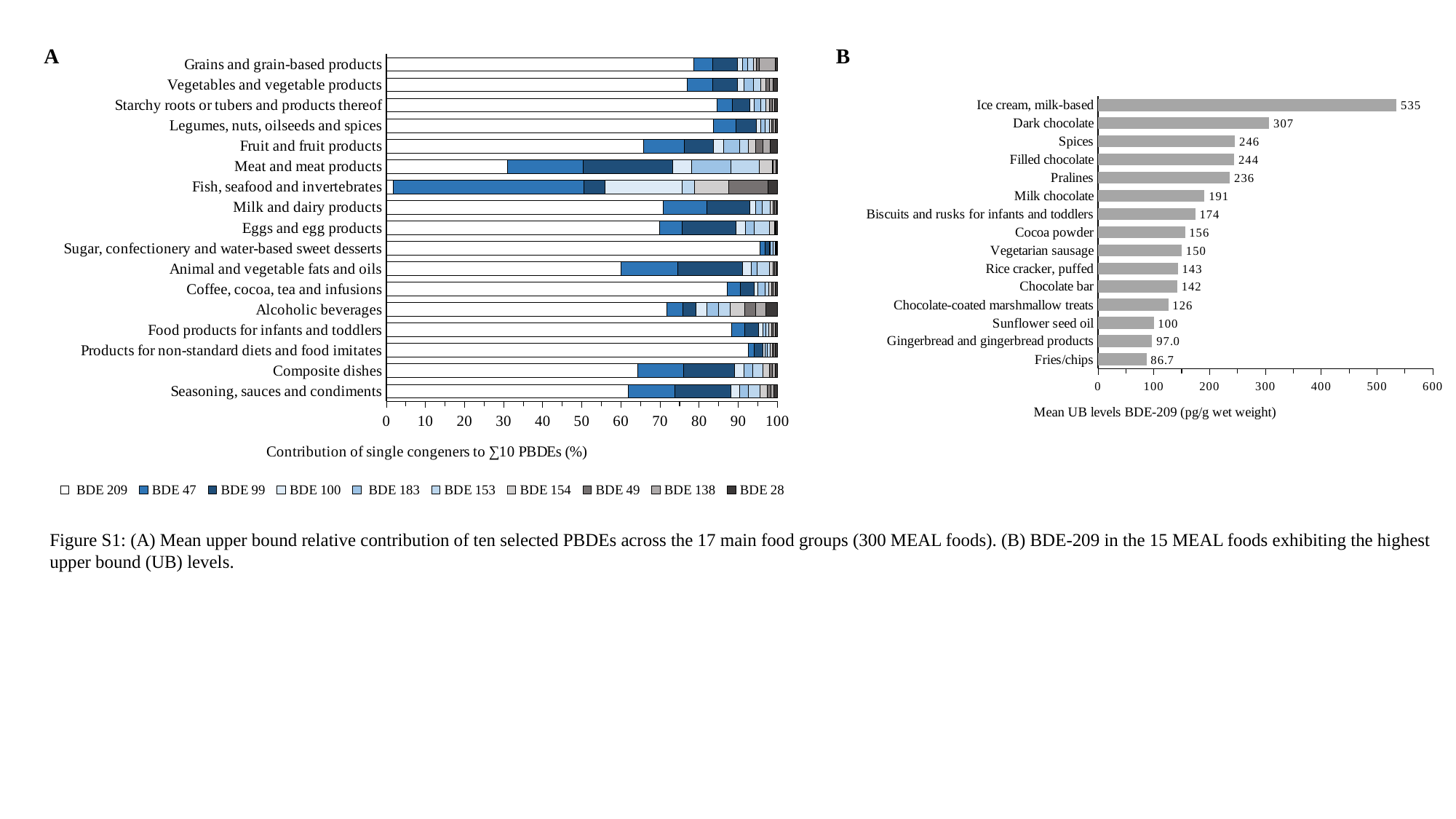

B
A
### Chart
| Category | BDE 209 | BDE 47 | BDE 99 | BDE 100 | BDE 183 | BDE 153 | BDE 154 | BDE 49 | BDE 138 | BDE 28 |
|---|---|---|---|---|---|---|---|---|---|---|
| Seasoning, sauces and condiments | 61.8582405875286 | 12.02118143886623 | 14.155889670997219 | 2.331213678313435 | 2.1664420308933763 | 3.087070918065772 | 1.806385468012505 | 0.8578587357742784 | 0.8578587357742784 | 0.8578587357742784 |
| Composite dishes | 64.3846794425165 | 11.673640036316858 | 13.030152103345616 | 2.424944272962254 | 2.1043337560126174 | 2.6964763683607367 | 1.6336298330164158 | 0.8112616768387029 | 0.6326195099103842 | 0.6082630007199337 |
| Products for non-standard diets and food imitates | 92.56542897944155 | 1.4957907452736583 | 2.2168884971076124 | 0.6146243303411827 | 0.6146243303411827 | 0.7307200371834062 | 0.4985286234989593 | 0.38243291665673596 | 0.38243291665673596 | 0.4985286234989593 |
| Food products for infants and toddlers | 88.31134932198889 | 3.37825776881861 | 3.541779354090436 | 1.0544794611514312 | 0.8214291348795277 | 0.7292304040580089 | 0.6571011542446706 | 0.5021244669228143 | 0.5021244669228143 | 0.5021244669228143 |
| Alcoholic beverages | 71.8232044198895 | 4.055483719289997 | 3.3384271776184327 | 2.7506759139532155 | 3.0092864699659114 | 2.9152462677794766 | 3.7145879863641715 | 2.7506759139532155 | 2.7506759139532155 | 2.891736217232867 |
| Coffee, cocoa, tea and infusions | 87.16258547441242 | 3.365599103438794 | 3.584961500688354 | 0.9016155173163817 | 1.9228168416776974 | 0.8358041656874485 | 0.7151500210344047 | 0.4913914254960328 | 0.4913914254960328 | 0.5286845247524281 |
| Animal and vegetable fats and oils | 60.10815714857001 | 14.366300339353435 | 16.65747324762405 | 2.2367144146231754 | 1.4275166379444668 | 3.236009691703766 | 0.9525824855454149 | 0.30702686004570573 | 0.30702686004570573 | 0.4011923145442597 |
| Sugar, confectionery and water-based sweet desserts | 95.5692422066202 | 1.335950740503685 | 1.0219657419591257 | 0.2582251512951571 | 0.796765351641014 | 0.41138530097032955 | 0.17910854383353514 | 0.13301529349361818 | 0.16811167700116922 | 0.12622999268215837 |
| Eggs and egg products | 69.94793290006389 | 5.772077593825313 | 13.744835141680115 | 2.339198853170954 | 2.1864827261396402 | 3.9403665998318917 | 1.2797622408334421 | 0.26311464815158125 | 0.26311464815158125 | 0.26311464815158125 |
| Milk and dairy products | 70.76916691145298 | 11.125101704173442 | 11.12642767560649 | 1.501864830510596 | 1.624220407762415 | 1.9711054374355872 | 0.7982476295037901 | 0.3540199424628449 | 0.36492273054593977 | 0.36492273054593977 |
| Fish, seafood and invertebrates | 1.6675263572956949 | 48.789826390267436 | 5.4035106802618165 | 19.73921404233811 | 0.1377474452628858 | 3.0664123353446744 | 8.724425310216862 | 10.07196849010025 | 0.020600570140344995 | 2.3787683787719214 |
| Meat and meat products | 31.02236912711693 | 19.29129202868625 | 22.972316862542055 | 4.712051822435466 | 10.190003802495548 | 7.216142812320416 | 3.2503780818831505 | 0.3230401689020052 | 0.6021862433034813 | 0.4202190503146923 |
| Fruit and fruit products | 65.81841708000862 | 10.38027460283229 | 7.476098051901372 | 2.6525770972611595 | 3.982459923801308 | 2.3290920854000428 | 1.8402702896987995 | 1.8402702896987995 | 1.8402702896987995 | 1.8402702896987995 |
| Legumes, nuts, oilseeds and spices | 83.71115893629005 | 5.735959209960898 | 5.139852598781031 | 1.0766057281293726 | 1.2396075869855288 | 1.0212804641051407 | 0.6550540839581033 | 0.39931801166966224 | 0.42026988265232973 | 0.6008934974679079 |
| Starchy roots or tubers and products thereof | 84.57129607326249 | 3.8537429284731815 | 4.499213211739467 | 1.150917019164316 | 1.6152233960887958 | 1.3901051527314725 | 0.8934380283243775 | 0.7034945104916358 | 0.6303310814005058 | 0.6922385983237695 |
| Vegetables and vegetable products | 76.8929650565274 | 6.655266027935661 | 6.2040799206430775 | 1.798050516689969 | 2.347161034141138 | 1.7646352791743418 | 1.3488869249114586 | 0.9409070293784316 | 0.9409070293784316 | 1.1071411812201177 |
| Grains and grain-based products | 78.55129040448571 | 4.9716731740333655 | 6.29960067850036 | 1.2511899154380626 | 1.414076509818369 | 1.3861803432862165 | 0.8325453125205483 | 0.5873632377521342 | 4.237029117686513 | 0.4690513064787152 |
### Chart
| Category | |
|---|---|
| Fries/chips | 86.7 |
| Gingerbread and gingerbread products | 97.0 |
| Sunflower seed oil | 99.825 |
| Chocolate-coated marshmallow treats | 126.0 |
| Chocolate bar | 142.0 |
| Rice cracker, puffed | 143.08333333333331 |
| Vegetarian sausage | 150.0 |
| Cocoa powder | 155.92311999999998 |
| Biscuits and rusks for infants and toddlers | 174.2 |
| Milk chocolate | 190.7625 |
| Pralines | 236.367 |
| Filled chocolate | 244.0 |
| Spices | 245.5 |
| Dark chocolate | 306.76 |
| Ice cream, milk-based | 535.0 |Figure S1: (A) Mean upper bound relative contribution of ten selected PBDEs across the 17 main food groups (300 MEAL foods). (B) BDE-209 in the 15 MEAL foods exhibiting the highest upper bound (UB) levels.
