# Supplementary material for: Germany's first Total Diet Study - Occurrence of non-dioxin-like polychlorinated biphenyls and polybrominated diphenyl ethers in foods
Source: Food Chem X. 2024 Mar 11;22:101274. doi: 10.1016/j.fochx.2024.101274 (PMC10957405; doi:10.1016/j.fochx.2024.101274)
Supplement: Fig. S2: Mean upper bound levels of the sum of six NDL-PCBs in MEAL food groups according to conventional and organic type of production based on the wet weight in (A) and on the fat content in (B). (C) Comparison of foods of animal origin by the type of production. 1 incudes liver samples. Data are [file mmc2.pptx]

## Slide 1
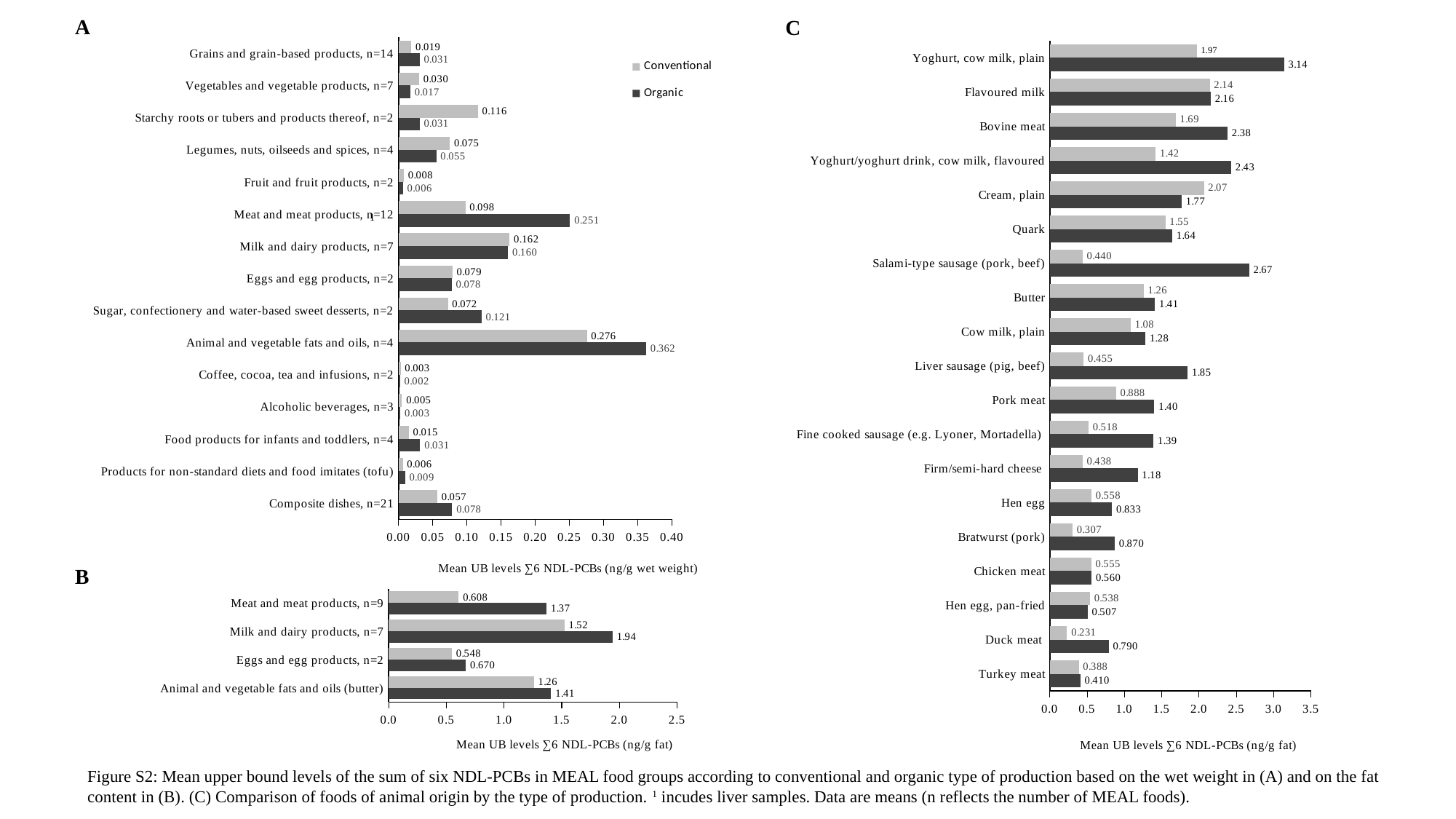

A
C
### Chart
| Category | Organic | Conventional |
|---|---|---|
| Composite dishes, n=21 | 0.07834007936507938 | 0.056529861111111114 |
| Products for non-standard diets and food imitates (tofu) | 0.0092 | 0.006 |
| Food products for infants and toddlers, n=4 | 0.0314475 | 0.0148275 |
| Alcoholic beverages, n=3 | 0.0026999999999999997 | 0.004733333333333333 |
| Coffee, cocoa, tea and infusions, n=2 | 0.00202 | 0.002875 |
| Animal and vegetable fats and oils, n=4 | 0.36231705882352944 | 0.2756789531672347 |
| Sugar, confectionery and water-based sweet desserts, n=2 | 0.12115000000000001 | 0.07225000000000001 |
| Eggs and egg products, n=2 | 0.07758931111983705 | 0.07885360071469683 |
| Milk and dairy products, n=7 | 0.16031168637915877 | 0.16248885363384893 |
| Meat and meat products, n=12 | 0.2508645303291574 | 0.09765155158482948 |
| Fruit and fruit products, n=2 | 0.006200000000000001 | 0.00755 |
| Legumes, nuts, oilseeds and spices, n=4 | 0.055099999999999996 | 0.0749375 |
| Starchy roots or tubers and products thereof, n=2 | 0.030574999999999998 | 0.116 |
| Vegetables and vegetable products, n=7 | 0.017121428571428572 | 0.02986428571428571 |
| Grains and grain-based products, n=14 | 0.030613471428571435 | 0.01858137142857143 |
### Chart
| Category | Organic | Conventional |
|---|---|---|
| Turkey meat | 0.41 | 0.388 |
| Duck meat | 0.79 | 0.231 |
| Hen egg, pan-fried | 0.5065000000000001 | 0.5382625 |
| Chicken meat | 0.56 | 0.5549999999999999 |
| Bratwurst (pork) | 0.87 | 0.3065 |
| Hen egg | 0.833 | 0.55755 |
| Firm/semi-hard cheese | 1.1776200000000001 | 0.43848 |
| Fine cooked sausage (e.g. Lyoner, Mortadella) | 1.39 | 0.5183 |
| Pork meat | 1.4 | 0.8875000000000001 |
| Liver sausage (pig, beef) | 1.8472250000000001 | 0.4545 |
| Cow milk, plain | 1.2800500000000001 | 1.0843 |
| Butter | 1.40825 | 1.2589 |
| Salami-type sausage (pork, beef) | 2.67 | 0.44 |
| Quark | 1.6400000000000001 | 1.55 |
| Cream, plain | 1.7668750000000002 | 2.065 |
| Yoghurt/yoghurt drink, cow milk, flavoured | 2.4299999999999997 | 1.42 |
| Bovine meat | 2.380925 | 1.68706875 |
| Flavoured milk | 2.15575 | 2.143 |
| Yoghurt, cow milk, plain | 3.14 | 1.97 |1
B
### Chart
| Category | Organic | Conventional |
|---|---|---|
| Animal and vegetable fats and oils (butter) | 1.40825 | 1.2589 |
| Eggs and egg products, n=2 | 0.6697500000000001 | 0.54790625 |
| Milk and dairy products, n=7 | 1.9414707142857144 | 1.5243971428571428 |
| Meat and meat products, n=9 | 1.3686833333333335 | 0.6075409722222224 |Figure S2: Mean upper bound levels of the sum of six NDL-PCBs in MEAL food groups according to conventional and organic type of production based on the wet weight in (A) and on the fat content in (B). (C) Comparison of foods of animal origin by the type of production. 1 incudes liver samples. Data are means (n reflects the number of MEAL foods).
