# Supplementary material for: Germany's first Total Diet Study - Occurrence of non-dioxin-like polychlorinated biphenyls and polybrominated diphenyl ethers in foods
Source: Food Chem X. 2024 Mar 11;22:101274. doi: 10.1016/j.fochx.2024.101274 (PMC10957405; doi:10.1016/j.fochx.2024.101274)
Supplement: Fig. S3: (A) Mean upper bound (UB) levels of the sum of nine PBDEs (without BDE-209) in MEAL food groups according to conventional and organic type of production and (B) mean UB levels of the sum of ten PBDEs (including BDE-209). Data are means (n reflects the number of MEAL foods). [file mmc3.pptx]

## Slide 1
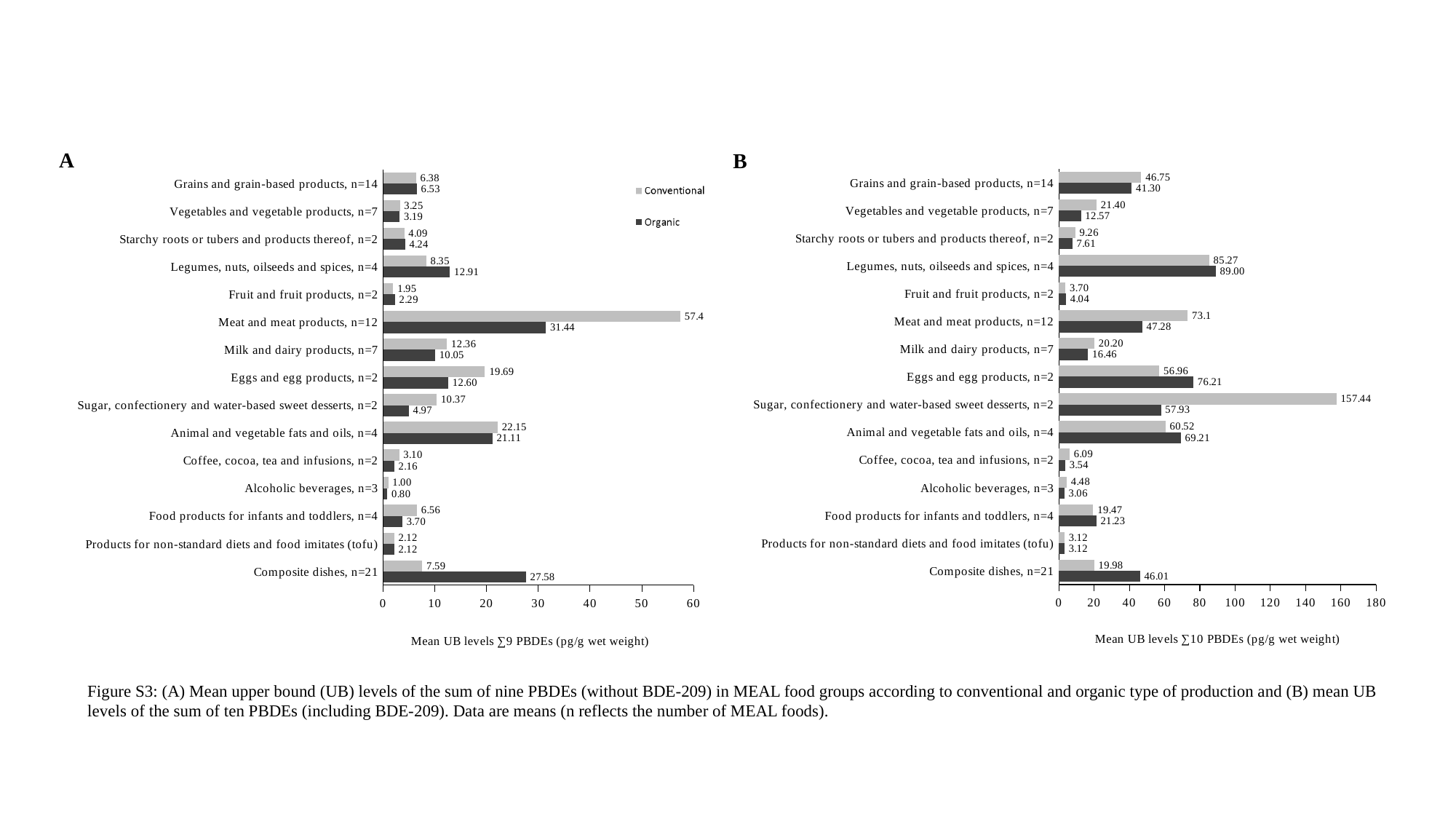

A
B
### Chart
| Category | | |
|---|---|---|
| Composite dishes, n=21 | 46.00591190476191 | 19.9842880952381 |
| Products for non-standard diets and food imitates (tofu) | 3.12 | 3.1199999999999997 |
| Food products for infants and toddlers, n=4 | 21.23345875 | 19.47037125 |
| Alcoholic beverages, n=3 | 3.0566666666666666 | 4.48 |
| Coffee, cocoa, tea and infusions, n=2 | 3.535 | 6.08975 |
| Animal and vegetable fats and oils, n=4 | 69.21058333333333 | 60.524649999999994 |
| Sugar, confectionery and water-based sweet desserts, n=2 | 57.927699999999994 | 157.43892499999998 |
| Eggs and egg products, n=2 | 76.21483333333333 | 56.95878333333333 |
| Milk and dairy products, n=7 | 16.455650000000002 | 20.198185714285717 |
| Meat and meat products, n=12 | 47.275305555555555 | 73.05992083333332 |
| Fruit and fruit products, n=2 | 4.04 | 3.7 |
| Legumes, nuts, oilseeds and spices, n=4 | 88.9956775 | 85.265515 |
| Starchy roots or tubers and products thereof, n=2 | 7.605 | 9.254999999999999 |
| Vegetables and vegetable products, n=7 | 12.567957142857143 | 21.400457380952382 |
| Grains and grain-based products, n=14 | 41.29950464285715 | 46.7457169047619 |
### Chart
| Category | | |
|---|---|---|
| Composite dishes, n=21 | 27.584749999999996 | 7.587933928571428 |
| Products for non-standard diets and food imitates (tofu) | 2.12 | 2.1199999999999997 |
| Food products for infants and toddlers, n=4 | 3.6987087499999998 | 6.560371250000001 |
| Alcoholic beverages, n=3 | 0.8000000000000002 | 0.9966666666666667 |
| Coffee, cocoa, tea and infusions, n=2 | 2.16 | 3.10475 |
| Animal and vegetable fats and oils, n=4 | 21.113500000000002 | 22.145899999999997 |
| Sugar, confectionery and water-based sweet desserts, n=2 | 4.9652 | 10.368925 |
| Eggs and egg products, n=2 | 12.598166666666668 | 19.693783333333332 |
| Milk and dairy products, n=7 | 10.053507142857143 | 12.362457142857144 |
| Meat and meat products, n=12 | 31.44169444444444 | 57.41805395833333 |
| Fruit and fruit products, n=2 | 2.29 | 1.9499999999999997 |
| Legumes, nuts, oilseeds and spices, n=4 | 12.9064675 | 8.3505075 |
| Starchy roots or tubers and products thereof, n=2 | 4.2425 | 4.0875 |
| Vegetables and vegetable products, n=7 | 3.1935523809523816 | 3.2454573809523803 |
| Grains and grain-based products, n=14 | 6.531473214285716 | 6.377210714285716 |
Figure S3: (A) Mean upper bound (UB) levels of the sum of nine PBDEs (without BDE-209) in MEAL food groups according to conventional and organic type of production and (B) mean UB levels of the sum of ten PBDEs (including BDE-209). Data are means (n reflects the number of MEAL foods).
