# Supplementary material for: Germany's first Total Diet Study - Occurrence of non-dioxin-like polychlorinated biphenyls and polybrominated diphenyl ethers in foods
Source: Food Chem X. 2024 Mar 11;22:101274. doi: 10.1016/j.fochx.2024.101274 (PMC10957405; doi:10.1016/j.fochx.2024.101274)
Supplement: Table S1: Mean levels of ∑6 NDL-PCBs in main food groups with foods of animal origin expressed in ng/g fat. [file mmc4.docx]

**Table S1**

Mean levels of ∑6 NDL-PCBs in main food groups with foods of animal origin expressed in ng/g fat.

| **Main food group** | **MEAL foods (n)** | **Mean** | **SD** | **Median** | **Minimum** | **Maximum** |
| --- | --- | --- | --- | --- | --- | --- |
| Meat and meat products^1^ | 28 | 1.19 | 2.16 | 0.568 | 0.044 | 11.8 (wild boar meat) |
| Milk and dairy products^2^ | 22 | 1.44 | 0.775 | 1.47 | 0.170 | 2.75 (yoghurt, cow milk, plain) |
| Eggs and egg products | 2 | 0.572 | 0.057 | 0.572 | 0.532 | 0.613 (hen egg) |
| Animal fats | 2 | 1.36 | 0.009 | 1.36 | 1.36 | 1.37 (butter, low fat) |
| Total/Mean | 54 | 1.14 | 0.751 | 0.994 |  |  |

Left-censored data were analysed using the upper bound scenario.

Results below the limit of quantification (LOQ) were set to the value reported as the LOQ.

∑6 NDL-PCBs: PCB 28, 52, 101, 138, 153, 180

^1^ does not include liver and edible offal

^2^ does not include buttermilk
