# Supplementary material for: Germany's first Total Diet Study - Occurrence of non-dioxin-like polychlorinated biphenyls and polybrominated diphenyl ethers in foods
Source: Food Chem X. 2024 Mar 11;22:101274. doi: 10.1016/j.fochx.2024.101274 (PMC10957405; doi:10.1016/j.fochx.2024.101274)
Supplement: Table S6: Mean levels of ∑6 NDL-PCBs in the main food groups with MEAL foods that were analysed for differences in the region (ng/g wet weight). [file mmc9.docx]

| **Table S6** |  |  |  |  |  |  |
| --- | --- | --- | --- | --- | --- | --- |
| Mean levels of ∑6 NDL-PCBs in the main food groups with MEAL foods that were analysed for differences in the region (ng/g wet weight). | | | | | |  |
| **Main food group** | **MEAL foods (n)** | **East** | **South** | **West** | **North** |  |
| Grains and grain-based products | 14 | 0.051 | 0.055 | 0.055 | 0.067 |  |
| Vegetables and vegetable products | 9 | 0.049 | 0.046 | 0.051 | 0.039 |  |
| Meat and meat products^1^ | 8 | 0.152 | 0.087 | 0.137 | 0.123 |  |
| Fish, seafood and invertebrates | 3 | 2.45 | 1.99 | 1.44 | 1.49 |  |
| Eggs and egg products | 2 | 0.091 | 0.044 | 0.124 | 0.056 |  |
| Composite dishes | 15 | 0.064 | 0.071 | 0.075 | 0.067 |  |
| **Total/Mean** | **51** | **0.477** | **0.381** | **0.314** | **0.307** |  |
| Meat and meat products (without liver and edible offal, ng/g fat) | 4 | 1.34 | 0.668 | 0.825 | 1.52 |  |
| Eggs and egg products (ng/g fat) | 2 | 0.610 | 0.338 | 0.854 | 0.390 |  |
| Left-censored data were analysed using the upper bound scenario.  Results below the limit of quantification (LOQ) were set to the value reported as the LOQ. | | | | | | |
| ∑6 NDL-PCBs: PCB 28, 52, 101, 138, 153, 180 |  |  |  |  |  |  |
| ^1^ includes liver and edible offal |  |  |  |  |  |  |
