# Supplementary material for: Germany's first Total Diet Study - Occurrence of non-dioxin-like polychlorinated biphenyls and polybrominated diphenyl ethers in foods
Source: Food Chem X. 2024 Mar 11;22:101274. doi: 10.1016/j.fochx.2024.101274 (PMC10957405; doi:10.1016/j.fochx.2024.101274)
Supplement: Table S7: Mean levels of ∑9 PBDEs and ∑10 PBDEs in main food groups with MEAL foods that were analysed for differences in the region (pg/g wet weight). [file mmc10.docx]

| **Table S7** |  |  |  |  |  |
| --- | --- | --- | --- | --- | --- |
| Mean levels of ∑9 PBDEs and ∑10 PBDEs in main food groups with MEAL foods that were analysed for differences in the region (pg/g wet weight). | | | | | |
| **Main food group** | **MEAL foods (n)** | ∑9 PBDEs (∑10 PBDEs) | | | |
|  |  | **East** | **South** | **West** | **North** |
| Grains and grain-based products | 14 | 9.50 (33.7) | 9.33 (40.0) | 9.89 (51.3) | 8.82 (35.8) |
| Vegetables and vegetable products | 9 | 4.89 (18.7) | 4.10 (16.1) | 4.20 (17.4) | 3.76 (14.4) |
| Meat and meat products^1^ | 8 | 10.4 (25.2) | 13.8 (27.8) | 7.50 (19.6) | 108 (119) |
| Fish, seafood and invertebrates | 3 | 107 (114) | 112 (120) | 111 (122) | 122 (136) |
| Eggs and egg products | 2 | 8.10 (30.2) | 42.3 (119) | 15.2 (49.6) | 13.2 (29.2) |
| Composite dishes | 15 | 10.9 (26.0) | 8.15 (27.3) | 10.2 (25.3) | 8.93 (23.2) |
| **Total/Mean** | **51** | **25.2 (41.2)** | **31.6 (58.4)** | **26.3 (47.6)** | **44.0 (59.6)** |

Left-censored data were analysed using the upper bound scenario.

Results below the limit of detection (LOD) and below the limit of quantification (LOQ) were set to the value reported as the LOD and the LOQ, respectively.

∑9 PBDEs: BDE-28, 47, 49, 99, 100, 138, 153, 154, 183

∑10 PBDEs: BDE-28, 47, 49, 99, 100, 138, 153, 154, 183, 209

^1^ includes liver and edible offal
